# Exploring Zanidatamab’s efficacy across HER2-positive Malignancies: a narrative review

**DOI:** 10.1186/s12885-025-13749-1

**Published:** 2025-03-01

**Authors:** Warisha Kanwal, Kaneez Narjis, Sarah Musani, Fnu Nancy, Laiba Qureshi, Muhammad Mudasir, Rohma Naseem, Fnu Tooba, Juvairia Yousuf, Kanza Farhan, Hadiya Javed, Mohammed Mahmmoud Fadelallah Eljack

**Affiliations:** 1 Karachi Medical and Dental College, Karachi, Pakistan; 2 Isra University of Medical Sciences, Hyderabad, Pakistan; 3 Dow University of Health Sciences, Karachi, Pakistan; 4 Jinnah Sindh Medical University, Karachi, Pakistan; 5 Liaquat University of Medical and Health Sciences, Jamshoro, Pakistan; 6 Liaquat National Hospital and Medical College, Karachi, Pakistan; 7 Hamdard University, Karachi, Pakistan; 8 Community Department , University of Bakhtalruda, Ad Duwaym, 1131 Sudan

**Keywords:** Zanidatamab, Bispecific antibody, Targeted therapy, HER2, Malignancies, Efficacy, Outcome

## Abstract

**Background:**

HER2-positive cancers involve amplification or overexpression of the HER2 gene, leading to aggressive tumor growth across several cancer types, including breast, gastric, ovarian, and pancreatic cancers. Detection methods such as immunohistochemistry, next-generation sequencing, and fluorescence in situ hybridization are used, with new advancements like biosensors and circulating tumor DNA offering improved diagnostic potential. Treatment strategies have evolved, including anti-HER2 drugs like trastuzumab and newer agents like zanidatamab, which show promise against HER2-positive malignancies.

**Methods:**

A comprehensive search of the following academic databases was performed including PubMed, Cochrane Library, and clinicaltrials.gov. A detailed search string was made. Studies were selected based on whether they contained the keywords and if they reported the details of treatment for zanidatamab. A total of 16 studies were selected. Abstracts were independently examined by one author and critically reviewed by another and if there were any conflicting viewpoints they were discussed until consensus was reached.

**Discussion:**

Zanidatamab has shown promising clinical outcomes in several HER2-positive cancers, including biliary tract, breast, gastric, and lung cancers, with high disease control rates and progression-free survival. Although it is not yet FDA-approved, it has received priority review for HER2-positive biliary tract cancer, with an FDA decision expected in November 2024.

The safety profile of zanidatamab has been well-studied. The most common side effects include diarrhea, infusion reactions, and other mild to moderate treatment-related adverse events. In combination with Palbociclib for HER2-positive breast cancer, more severe side effects were observed, resulting in some patients discontinuing treatment. However, no treatment-related deaths have been reported across trials.

While its anticancer efficacy and manageable safety profile are promising, long-term safety and efficacy data are still needed. Early clinical trials demonstrate strong efficacy, though some side effects, such as diarrhea and decreased ejection fraction, were noted. Future research should focus on understanding potential resistance mechanisms and establishing zanidatamab’s broader role in the treatment landscape of HER2-positive cancers.

**Conclusion:**

In summary, zanidatamab has shown significant tumor response, progression-free survival, disease control, and improved quality of life in early trials, however, the lack of long-term safety and efficacy data remains a key limitation, requiring further research.

**Supplementary Information:**

The online version contains supplementary material available at 10.1186/s12885-025-13749-1.

## Background

Receptor tyrosine-protein kinase, commonly known as Human Epidermal Growth Factor Receptor 2 (HER2) or CD340, is located on the surfaces of cells. This protein is a member of the Epidermal Growth Factor Receptor (EGFR) family and is encoded by the Erythroblastic oncogene B (ERBB2) gene. The ERBB2 gene is vital for normal processes such as cell division, differentiation, and survival. Through various signaling pathways, HER2 receptors promote cell proliferation and inhibit apoptosis (programmed cell death). It is crucial to regulate these receptors to prevent uncontrolled cell growth. Cancers that overexpress or amplify the HER2 gene are referred to as HER2-positive malignancies [1]. This gene serves as an important biomarker and is a key target for drug therapy.

However, when HER2 is amplified or overexpressed, unchecked cell proliferation and division accelerate the progression of cancer [1]. This amplification or overexpression is usually found across various tumor types and could have important therapeutic significance for malignancies that are not usually expected to respond well to anti-HER2 treatments [2].

HER2-positivity is found in various types of malignancies like gastric, biliary tract, breast, esophageal, ovarian, and uterine cancers [2]. The incidence, description, and treatment trends for individual HER2-positive tumors are present in Table 1. Recently, an association has been discovered with pancreatic and biliary tract cancers (BTC) [3]. Patients with HER2-positive cancers tend to have poorer prognosis compared to those with HER2-negative cancers, as these malignancies are more aggressive. However, new treatment strategies have shown promising results [4].

**Table 1:** Incidence and description of different HER2-positive tumors

**Name of tumor**	**Incidence of HER2-positive tumors **[5–10]	**Description**
Endometrial cancer	17–30%	HER2 overexpression is seen in 60%-70% of high-grade carcinomas, 17%–33% of carcinosarcoma, uterine serous carcinoma, and a subset of high-grade endometrioid endometrial tumors Anti-HER2-targeted therapies like trastuzumab and lapatinib are considered for treatment due to the expression of HER2 in certain cancers. However, treatment with trastuzumab has not shown any responses in women with HER2-expressing endometrial cancer. Clinical trials indicate that these therapies have low clinical effectiveness, suggesting that tumors may have acquired or innate mechanisms of resistance to trastuzumab. While these resistance mechanisms are not fully understood, ongoing research aims to explore alternative treatment options [10] Zanidatamab is being tested for HER2-positive endometrial cancers in various clinical trials [11]
Gastric cancer	22%	Heterogeneous HER2 expression is commonly observed in gastric tumors. HER2-positive gastric tumors are associated with decreased survival rates and complications such as serious invasion and metastasis to distant sites. Anti-HER2 therapies, including trastuzumab, have been shown to improve survival rates. Other agents, such as pertuzumab, lapatinib, zanidatamab, and afatinib, are currently being tested. The treatment of HER2-positive tumors differs from that of other HER2-expressing tumors, as it requires HER2 testing through immunohistochemistry and a specific scoring system [12]
Biliary tract cancer	5–20%	BTCs include intrahepatic cholangiocarcinoma (IHCh), extrahepatic cholangiocarcinoma (ECC), gallbladder cancer (GBC), and ampulla of Vater cancer. HER2 overexpression is observed in 38–100% of iCCAs and in 5–15% of ECCs and GBCs, as demonstrated in the HERIZON-BTC-01 trial In HER2-positive BTCs, HER2 inhibitors have been tested both as monotherapy and in combination therapies. These drugs have shown satisfactory results when used as first-line treatments. However, drugs such as erlotinib, cetuximab, and panitumumab have failed to demonstrate efficacy when evaluated as second-line therapies. Recently, zanidatamab has been assessed in HER2-positive BTCs, with its application noted in 5–19% of these cancers [13]
Breast cancer	14%	HER2 overexpression has been identified in various breast cancer types. Approximately 40% of ductal carcinoma in situ and 15–30% of invasive breast cancers exhibit HER2 receptor overexpression, which aids in the diagnosis and treatment of these cancers [14] Advanced breast cancers that overexpress HER2 are clinically aggressive tumors associated with a poor prognosis. Surgical resection, along with chemotherapeutic agents like trastuzumab and pertuzumab, are the first-line treatment options approved by the FDA and the European Medicines Agency (EMA). T-DM1, an antibody–drug conjugate that specifically targets HER2-overexpressing cells, is used as second-line or third-line therapy for breast cancers [15] Zanidatamab, in combination with docetaxel, is being used in first-line settings for advanced breast cancers and has shown satisfactory results so far [16]
Esophageal cancer	8.6–10%	Aggressive esophageal cancer often demonstrates HER2 expression. While surgical resection is the standard treatment, anti-HER2 drugs such as Trastuzumab, Ramucirumab, Entrectinib, Larotrectinib, and Zolbetuximab are recommended for cancers located at the gastric and esophageal junction. Anti-HER2 therapy has been shown to improve survival rates [17] Zanidatamab in combination with chemotherapy is being evaluated in ongoing trials [18]
Ovarian cancer	6.6–39.2%	HER2 expression has been identified in clear-cell ovarian carcinomas and mucinous ovarian cancers [19] Studies show that HER2 expression is linked to poor prognosis but does not affect overall survival (OS) [20]

These malignancies predominantly affect older adults, particularly females, who are postmenopausal and of Asian or Caucasian descent, due to their association with breast, uterine, and ovarian cancers [21]. However, HER2-positive cancers can also affect younger individuals and men [21]. HER2-positive malignancies include various types of cancer that require specific detection methods and assessment tools. The FDA has approved several drugs for treating HER2-positive cancers, including trastuzumab, ado-trastuzumab emtansine (T-DM1), and pertuzumab. Additionally, there are two FDA-approved small-molecule tyrosine kinase inhibitors (TKIs): lapatinib and afatinib. Several other treatments, such as erlotinib, Ontruzant, and zanidatamab, are currently being evaluated [15].

HER2 malignancies encompass various cancer types, requiring specific detection methods and assessment tools. For breast cancer, techniques such as mammography, ultrasound, and molecular bioimaging are utilized [22]. In the case of gynecological cancers, tru-cut biopsies are commonly performed [23], while biliary tract biopsies are used for detecting HER2-positive gastrointestinal cancers [3]. However, recent advances in several diagnostic techniques have improved their capacity to detect HER2 mutations; these techniques include immunohistochemistry (IHC), next-generation sequencing (NGS), and fluorescent in situ hybridization (FISH) [24].

IHC is typically the first method used and assesses the number of HER2 receptors on the cell surface, with results ranging from 0 to + 3. A score of 0 to + 1 indicates a negative test outcome, + 3 correlates to a positive test outcome, and + 2 corresponds to an indefinite result that is further confirmed using NGS or FISH [25]. NGS and FISH quantify the number of HER2 receptors in the nucleus of mutated tumor cells. 44% of HER2 over expressive endometrial cancers are detected using IHC and 12% are detected using FISH [26].

Recent advancements involve testing with biomarkers CA such as CA-15–3, CA 27–29, HER2, and circulating tumor cells (CTC) in fluids. These assessment tools enable examining minute details such as the lipid composition of the membrane [27]. CTC and circulating tumor DNA (CTD) have the potential to revolutionize disease detection and prognosis [28].

Alternatively, biosensors based on parameters, nanomaterials, and antibodies have also become a modality for rapid and accurate detection of the HER2-extracellular domain. Biosensors couple the specificity and affinity of antibodies and conductive properties of nanometers [29]. Protein expression and gene amplification are also suggested methods for future trials [26].

Neoadjuvant therapy involving trastuzumab and chemotherapy is the preferred treatment for advanced gastric cancer [30]. In recent years, there has been a development of antibody–drug conjugates for therapeutic purposes [31]. Liquid biopsy-guided treatment is also being investigated in several trials. While breast cancer cells are sensitive to trastuzumab and lapatinib, endometrial cancers have shown resistance to trastuzumab suggesting that these tumors possess either innate or acquired resistance [32].

To counter HER2 overexpression in malignancies, several treatment modalities have emerged, including anti-HER2 drugs such as monoclonal antibodies, small molecule inhibitors, and antibody–drug conjugates. One such drug is zanidatamab, a novel bispecific antibody targeting two non-overlapping HER2 epitopes. It clusters HER2 receptors and induces cell death after internalization [33]. This review aims to address the potential of this drug and its probable expected outcomes in countering HER2-positive malignancies.

## Methods

A comprehensive search of the following academic databases was performed including PubMed, Cochrane Library, and clinicaltrials.gov. For finding the articles we used the following search string, (((zanidatamab[tiab]) OR ("ZW25"[tiab])) AND ((("HER2-positive"[tiab]) OR ("HER2 amplified"[tiab])) OR ("HER2 overexpressed"[tiab]))) AND ((((((malignancy[tiab]) OR (malignancies[tiab])) OR (cancer[tiab])) OR (tumor[tiab])) OR (carcinoma[tiab])) OR (neoplasm[tiab])).

Studies were selected based on whether they contained the keywords zanidatamab, HER2 positive tumors, and if they reported the details of treatment for zanidatamab. There were no limits to publication dates. All duplicate studies were excluded. A total of 16 studies were selected. The PRISMA flowchart is provided in the supplementary files. Abstracts were obtained and screened. Abstracts were independently examined by one author and critically reviewed by another and if there were any conflicting viewpoints they were discussed until consensus was reached.

### Zanidatamab

Zanidatamab (Zani/ZW25) was initially introduced by Zymeworks, which is currently being developed by BeiGene, Ltd. and Jazz Pharmaceuticals [34]. Zanidatamab is a HER2 antibody that has demonstrated anticancer effects in a variety of solid tumors that are HER2-amplified or express HER2 [35]. Numerous clinical trials are currently testing zanidatamab; one that particularly caught our attention was HERIZON-BTC-01, which produced important findings on HER2-amplified BTC [36].

Zanidatamab has not received FDA approval for use as a cancer treatment as of May 2024. The FDA has examined and granted priority review to the Biologics License Application (BLA) for zanidatamab, which is intended to treat HER2-positive BTC that has already been treated but is unresectable, locally progressed, or metastatic. November 29, 2024, is the date that the FDA has set under the Prescription Drug User Fee Act (PDUFA). Zanidatamab, if licensed, would be the first HER2-targeted medication authorized for BTC in the United States [37]. Zanidatamab is regarded by the FDA as a breakthrough treatment for HER2 gene-amplified BTC. The FDA recognizes zanidatamab's potential for two indications: one as a therapy for refractory BTC, and the other as a combination therapy for gastro-esophageal cancer (GEA) and routine chemotherapy. Zanidatamab has acquired Orphan Drug designation from the FDA and the European Medicine Agency for the treatment of BTC, GEA, and gastric cancers [37].

### Mechanism of action

HER2 receptors are a collection of proteins distributed throughout the body's cells. They are found mostly on epithelial surfaces, where they bind to numerous growth factors and transfer signals into the cell via the signal transduction pathways. These cells perform several roles such as cell growth regulation, motility, adhesion, epithelial proliferation, and differentiation [38]. Overexpression and mutations in the HER receptor family generate aberrant signaling, which leads to uncontrolled cell proliferation and survival, resulting in cancer initiation and progression. HER2 amplification or overexpression is seen in breast, gastric/gastroesophageal, and other cancers such as ovary, gallbladder, endometrial, bladder, biliary tract, lung, colon, and head and neck [39].

Various anti-HER2 agents, including trastuzumab, pertuzumab, lapatinib, neratinib, and many others have been approved for the treatment of HER2-positive breast cancer. Other solid tumors such as BTC, colorectal cancer, pancreatic cancer, and ovarian cancer, also show HER2 overexpression, but HER2 therapies have yet to have achieved approval in those cancers.

**Fig. 1:**
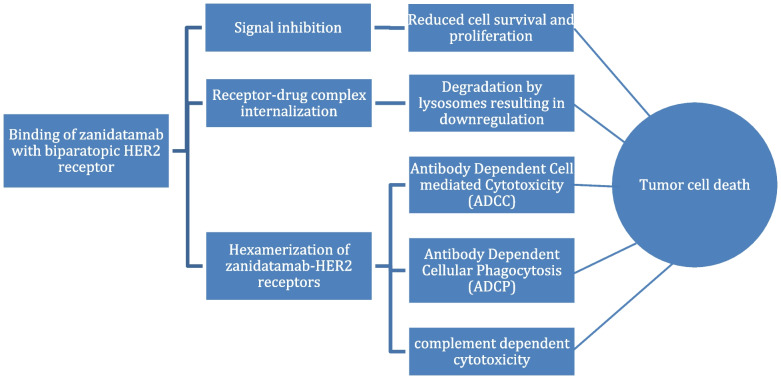
Mechanism of Action of Zanidatamab

Zanidatamab is an anti-HER2 IgG1 bispecific and biparatopic antibody (Ab) [34]. Zanidatamab is an IgG1-like antibody target and binds to two different binding domains ECD4 (juxtamembrane) and ECD2 (dimerization) extracellular domains on HER2 receptor at the same time [40]. Zanidatamab demonstrates antitumor activity through several mechanisms in tumors that express HER2, as illustrated in Fig. 1. These mechanisms include the inhibition of cell signaling, the internalization of the receptor-drug complex, and the hexamerization of zanidatamabs-HER2 receptors [41]. The hexamerization process leads to the induction of a cytotoxic T-lymphocyte (CTL) response as well as antibody-dependent cell-mediated cytotoxicity (ADCC) against tumor cells that overexpress HER2. Antibody-dependent cellular phagocytosis (ADCP) is also activated, stimulating the immune system to eliminate these tumor cells [42].

The uptake of HER2 receptors by the cells also suppresses HER2 activation, HER2-mediated signaling, and tumor cell proliferation. In summary, zanidatamab functions through multiple and unique mechanisms of action carried by the structural effects of biparatopic HER2 engagement [39].

### Adverse effects

Except for breast or GEA, the most common side effects observed for zanidatamab therapy on overexpressing or amplified HER2 malignancies were diarrhea (52%), infusion reactions (43%), and treatment-related adverse events (TRAE) of grade 1–3 (3%) [43]. The HERIZON-BTC-01 trial found 5% fewer adverse effects, including diarrhea, and a 3% fall in ejection fraction. There were no significant TRAE or treatment-related deaths [44].

The zanidatamab plus Palbociclib and Fulvestrant trial for HER2-positive metastatic breast cancer found moderate side effects such as diarrhea, neutropenia, nausea, stomatitis, anemia, vomiting, and asthenia [45]. Multiple patients reported severe TRAE such as neutropenia, diarrhea, anemia, thrombocytopenia, hypokalemia, and hypomagnesemia, as well as one serious adverse effect involving elevated transaminases.

Treatment was stopped because of these severe side effects, particularly asthenia, and only a small number of patients had their dosage reduced as a result. There were no treatment-related deaths, although there were 14 recorded deaths: 12 from disease progression, 1 from COVID-19 medication, and 1 from an idiopathic death for which the cause was unknown [46].

In the HER2-positive breast cancer trial, a considerable proportion of patients experienced grade 1–3 TRAE when zanidatamab and docetaxel were used as first-line therapy. Neutropenia and leukopenia were the most frequent side effects associated with the treatment. However, no documented deaths linked to therapy were found [47]. Various adverse events reported by clinical trials are present in Table 2.

**Table 2:** Adverse Events in different Clinical Trials

**Author/Unique Protocol Id; Phase; Trial No**	**Adverse Effects (AE)**
**Harding et al. (HERIZON-BTC-01); phase IIb; NCT04466891** [44]	Diarrhea (37%), infusion-related reaction (33%), decreased ejection fraction (3%)
**ZWI-ZW25-202, phase II; NCT04224272** [46]	Diarrhea (80%), neutrophil count decrease/neutropenia (59%), nausea (39%), stomatitis (37%), anemia (29%), vomiting (25%), and asthenia (24%)
**Wang X;phase Ib/II; NCT04276493** [47]	TRAE (97%, 67.5% ≥ grade 3 TRAE’s), neutropenia, leukopenia
**Lee et al.; Phase 1b/2; NCT04276493** [48]	Diarrhea, nausea, decreased appetite, peripheral sensory neuropathy, pyrexia, hypokalemia, Palmar-plantar erythrodysesthesia syndrome, fatigue, stomatitis, weight decrease
**Meric- Bernstam et al.; phase I; NCT02892123** [49]	Diarrhea (65%), nausea (45%) infusion reactions (37.1%) peripheral neuropathy (35%), and fatigue (30%)

*TRAE* Treatment-Related Adverse Effects

## Clinical Trials

### Primary submission / Completed studies (Table 3)

**Table 3:** Baseline Characteristics of completed trials or the primary submission of trials

Author/Unique Protocol Id; Phase; Trial No	Drugs	Zanidatamab Dose	Tumors Included	Number of Patients	Investigations
**Harding et al. (HERIZON-BTC-01); phase IIb; NCT04466891** [44]	Zanidatamab	20 mg/kg IV every 2 weeks	locally advanced or metastatic, HER2 + ve BTC (IHCh, ECC and GBC)	87	IHC
**Lumish et al.; phase II; NCT04513665** [50]	Zanidatamab	20 mg/kg IV every 2 weeks	recurrent or persistent HER2 + ve endometrial carcinoma and carcinosarcoma	16	IHC, FISH
**Lee et al.; Phase 1b/2; NCT04276493** [48]	Zanidatamab + chemotherapy / Zanidatamab + chemotherapy + Tislelizumab	Cohort A: 30 mg/kg IV, Cohort B: 1800 mg IV (weight < 70 kg) or 2400 mg IV (weight ≥ 70 kg)	unresectable, locally advanced, recurrent, or metastatic HER2 + ve Breast Cancer or Gastric cancer or GEJA	71	IHC, FISH
**ZWI-ZW25-202, phase II; NCT04224272** [46]	Zanidatamab + Palbociclib + Fulvestrant	NR	unresectable, locally advanced, or metastatic disease HER2 + ve breast cancer	51	NR

*BTC* Biliary Tract Cancer, *IHCh* Intra-hepatic Cholangiocarcinoma, *ECC* Extra-hepatic Cholangiocarcinoma, *GBC* Gall Bladder Cancer, *GEA* Gastroesophageal Adenocarcinoma, *NSCLC* Non-Small Cell Lung Carcinoma

#### Ongoing studies

There are 4 studies which are either completed or done with primary completion in Tables 3 and 4 and 12 ongoing studies evaluating zanidatamab for different cancers mentioned in Tables 5 and 6. In every trial, patients were investigated for malignancies by ISH or ISH and FISH. These clinical trials provide significant clinical insights into the efficacy along safety of zanidatamab, proposing valuable evidence that enlightens its potential role in clinical practice.

**Table 4:** Outcomes of completed trials or the primary submission of trials

Author/Unique Protocol Id; Phase; Trial No	Primary Outcome Measures	Secondary Outcome Measures	Adverse Effects (AE)	Incidence of AE	Incidence of Immune-Mediated AE	% of Patients with Grade 3 TRAE	% of Patients with Grade 4 TRAE	Treatment-Related Deaths	DLTs	ORR	CBR	DCR	DoR	PFS	OS	PCR
**Harding et al. (HERIZON-BTC-01); phase IIb; NCT04466891** [44]	ORR	DoR, DCR, PFS, OS, incidence of AE	diarrhea (37%), infusion-related reaction (33%), decreased ejection fraction (3%)	72%	NR	18%	none	None	NR	41.3% [95% CI 30·4–52·8]	NR	69 (95% CI;57- 79)	12.9 months (95% CI: 5.95- NR)	NYR	NYR	NR
**Lumish et al.; phase II; NCT04513665** [50]	ORR	none	NR	NR	NR	NR	NR	NR	NR	6.20%	37.50%	NR	NR	6.2 months	NR	NR
**Lee et al.; Phase 1b/2; NCT04276493** [48]	ORR, TRAE	DoR, PFS, OS, DCR	diarrhea, nausea, decreased appetite, peripheral sensory neuropathy, pyrexia, hypokalemia, Palmar-plantar erythrodysesthesia syndrome, fatigue, stomatitis, weight decrease	97.30%	27.30%	67.60%	none	none	NR	90.9% (95%CI: 75.7, 98.1)	NR	100%	16.4 months	10.9 months (95% CI: 6.9-NR)	NYR	NR
**ZWI-ZW25-202, phase II; NCT04224272** [46]	Grade 3 or Higher TRAE, PFS, DLTs	ORR, DoR, DCR, PFS, OS	diarrhea (80%), neutrophil count decrease/neutropenia (59%), nausea (39%), stomatitis (37%), anemia (29%), vomiting (25%), and asthenia (24%)	100%	present but not calculated	present but not calculated	none	None	12.5%	35% (95% CI; 21–50)	NR	91% (95% CI; 79–98)	15 months (95% CI, 12–25)	66.7% for 6 months [12 months (95% CI, 8–15) for all patients]	NYR	NR

*TRAE* Treatment-Related Adverse Effects, *DLTs* Dose Limiting Toxicities, *ORR* Objective response rate, *CBR* Clinical Benefit Rate, *DCR* Disease Control Rate, *DoR* Duration of Response, *PFS* Progression-Free Survival, *OS* Overall Survival, *pCR* Pathologic Complete Response, *NR* Not Reported, *NYR* Not Yet Recorded

**Table 5:** Baseline Characteristics of Ongoing Studies

Author/Unique Protocol Id; Phase; Trial No	Drugs	Zanidatamab Dose	Tumors Included	Number of Patients	Investigations
**Meric-Bernstam et al.; phase I; NCT02892123** [49]	Zanidatamab + chemotherapy	5 mg/kg to 30 mg/kg every 1, 2, or 3 weeks	locally advanced or metastatic, unresectable HER2 + ve tumors, received all available approved therapies, BTC, colorectal cancer, breast cancer, ovarian cancer, GEA, NSCLC	132	IHC, FISH
**Tabernero et al.; phase III; NCT05152147** [51]	Zanidatamab + chemotherapy / trastuzumab + chemotherapy / Zanidatamab + chemotherapy + tislelizumab	1,800 mg (patients < 70 kg at baseline) or 2,400 mg (patients ≥ 70 kg at baseline), intravenously on day 1 of each cycle	locally advanced or metastatic, unresectable, nonresponsive to chemoradiationHER2 + ve GEA (gastroesophageal functional tumor, gastric neoplasms, and esophageal adenocarcinoma)	714	IHC, ISH
**Elimova et al.; phase II; NCT06043427** [52]	Zanidatamab + Paclitaxel and Ramucirumab	assigned at enrollment	metastatic or unresectable HER2 + ve GEA (stomach, gastroesophageal junction, or esophagus)	168	IHC, FISH
**Garfin et al.; phase II; NCT03929666** [53]	Zanidatamab + chemotherapy	NR	locally advanced, recurrent, or metastatic, unresectable HER2 + ve GEA, BTC (ICC, ECC, and GBC), and colorectal cancer	74	IHC, FISH
**JZP598-303; phase III; NCT06435429** [54]	Zanidatamab + chemotherapy	NR	unresectable or metastatic, HER2 + ve breast cancer	550	NR
**JZP598-302; phase III; NCT06282575** [55]	Zanidatamab + Cisplatin ± PD-1/L1 inhibitor	NR	Locally advanced unresectable or metastatic HER2 + ve BTC (ICC, ECC, and GBC)	286	IHC, ISH
**RHA et al.; phase II; NCT05270889** [56]	Zanidatamab and tislelizumab	1800 mg IV (weight < 70 kg) or 2400 mg IV (weight ≥ 70 kg)	advanced HER2 + ve gastric cancer or GEJA after first-line treatment	50	IHC, FISH/SISH
**Hurvitz et al.; phase 1b/2; NCT05027139** [57]	Zanidatamab + Evorpacept	not provided	unresectable, locally advanced, or metastatic HER2 + ve breast, HER2 + ve breast cancer and HER2 overexpressing breast cancer cancer	52	NR
**Valero et al.; phase II; NCT05035836** [58]	Zanidatamab	every 2 weeks (± 3 days) for up to 6 doses	early stage, low-risk HER2 + ve breast cancer	20	NR
**Pohlmann et al.; phase I; NCT05868226** [59]	Zanidatamab + Tucatinib	NR	Metastatic HER2 + ve Breast Cancer	54	NR
**David et al.; phase III; NCT05615818** [60]	Zanidatamab + Futibatinib + Ivosidenib + Trastuzumab + Neratinib + Encorafenib + Binimetinib + Niraparib	1800 mg IV (weight < 70 kg) or 2400 mg IV (weight ≥ 70 kg)	De novo or recurrent, locally advanced unresectable or metastatic intrahepatic, HER2 + ve perihilar or distal cholangiocarcinoma, or GBC (ampullary carcinoma excluded)	800	NR
**BGB-A317-290-LTE1; phase III; NCT04164199** [48]	Zanidatamab or Tislelizumab or Pamiparib or Sitravatinib or BGB-15025 and others	NR	advanced malignancies	300	NR

*BTC* Biliary Tract Cancer, *ECC* Extra-hepatic Cholangiocarcinoma, *GBC* Gall Bladder Cancer, *GEA* Gastroesophageal Adenocarcinoma, *GEJA* Gastroesophageal Junctional Adenocarcinoma, *NSCLC* Non-Small Cell Lung Carcinoma

**Table 6:** Outcomes of Ongoing Studies

Author/Unique Protocol Id; Phase; Trial No	Primary Outcome Measures	Secondary Outcome Measures	Adverse Effects (AE)	Incidence of AE	Incidence of Immune-Mediated AE	% of Patients with Grade 3 Trae	% of Patients with Grade 4 TRAE	Treatment-Related Deaths	DLTs	ORR	CBR	DCR	DoR	PFS	OS	PCR
**Meric- Bernstam et al.; phase I; NCT02892123** [49]	safety and tolerability assessments	ORR, DCR, PFS	diarrhea (65%), nausea (45%) infusion reactions (37.1%) peripheral neuropathy (35%), and fatigue (30%)	NR	NR	4%	none	none	NR	37.8% (95% CI 27·0–48·7)	50%	81.3%	NR	NR	NR	NR
**Tabernero et al.; phase III; NCT05152147** [51]	PFS, OS	ORR, DoR, incidence of AE	NYR	NYR	NR	NR	NR	NR	NR	NYR	NR	NR	NYR	NYR	NYR	NR
**Elimova et al.; phase II; NCT06043427** [52]	PFS	OS, ORR, incidence of AE,	NYR	NYR	NR	NR	NR	NR	NR	NYR	NR	NR	NR	NYR	NYR	NR
**Garfin et al.; phase II; NCT03929666** [53]	DLTs, incidence of adverse events, ORR	ORR, DCR, CBR, DoR, PFS, OS	NYR	NYR	NR	NR	NR	NR	NYR	NYR	NYR	NYR	NYR	NYR	NYR	NR
**JZP598-303; phase III; NCT06435429** [54]	PFS	ORR, DoR, OS, TRAE, AE	NYR	NYR	NR	NYR	NYR	NR	NR	NYR	NR	NR	NYR	NYR	NYR	NR
**JZP598-302; phase III; NCT06282575** [55]	PFS	OS, PFS, ORR, DoR, TRAE	NR	NR	NR	NYR	NYR	NR	NR	NYR	NR	NR	NYR	NYR	NYR	NR
**RHA et al.; phase II; NCT05270889** [56]	ORR	none	NYR	NR	NR	NR	NR	NR	NR	NYR	NR	NR	NR	NR	NR	NR
**Hurvitz et al.; phase 1b/2; NCT05027139** [57]	ORR, incidence of adverse events, DLTs	DCR, CBR, DoR, PFS, OS	NYR	NYR	NR	NR	NR	NR	NYR	NYR	NYR	NYR	NYR	NYR	NYR	NR
**Valero et al.; phase II; NCT05035836** [58]	pCR	none	NR	NR	NR	NR	NR	NR	NR	NR	NR	NR	NR	NR	NR	NYR
**Pohlmann et al.; phase I; NCT05868226** [59]	incidence of adverse events, DLTs, ORR, DoR	PFS, CBR	NYR	NYR	NR	NR	NR	NR	NYR	NYR	NYR	NR	NYR	NYR	NR	NR
**David et al.; phase III; NCT05615818** [60]	PFS	OS, ORR, DoR, DCR, incidence of AE	NYR	NYR	NR	NR	NR	NR	NR	NYR	NR	NYR	NYR	NYR	NYR	NR
**BGB-A317-290-LTE1; phase III; NCT04164199** [48]	immune-mediated adverse events	OS	NR	NR	NYR	NR	NR	NR	NR	NR	NR	NR	NR	NR	NYR	NR

*TRAE* Treatment-Related Adverse Effects, *DLTs* Dose Limiting Toxicities, *ORR* Objective response rate, *CBR* Clinical Benefit Rate, *DCR* Disease Control Rate, *DoR* Duration of Response, *PFS* Progression-Free Survival, *OS* Overall Survival, *pCR* Pathologic Complete Response, *NR* Not Reported, *NYR* Not Yet Recorded

In a study done by Lee et al. (NCT04276493) evaluating zanidatamab + chemotherapy ± tocilizumab for breast, gastric, and gastroesophageal junctional adenocarcinoma, they concluded an exceptional Disease Control Rate (DCR) of 100% for median Duration of Response (DoR) of 16.4 months, median Objective Response Rate (ORR) of 90.9% and progression-free survival (PFS) 10.9 months. These promising results prove the efficacy and safety of zanidatamab for breast cancers and GEA [48].

In an ongoing study by Meric-Bernstam et al. (NCT02892123), remarkable results are recorded while the investigation of zanidatamab for multiple HER2-positive malignancies including BTC, colorectal, breast and ovarian cancers, GEA, and non-small cell lung carcinoma (NSCLC). He documented a significant DCR of 81.3%, a reasonable Clinical Benefit Rate (CBR) of 50%, and an ORR of 37.8%. This demonstrates that zanidatamab is highly effective in controlling the disease either by stabilizing it or by partial or complete response [49].

Harding et al. (NCT04466891) in a trial named HERIZON-BTC-01 investigated zanidatamab for BTC and documented a DCR of 69% for DoR of 12.9 months (95% CI: 5.95- NR) and median ORR of 41.%. It concludes that zanidatamab has rapid and long-lasting responses in patients with treatment-refractory BTC [36].

In the primary submission of a trial, NCT04224272, investigating zanidatamab in the treatment of breast malignancies, it recorded a significant DCR of 91% (95% CI; 79–98) and PFS 66.7% for 6 months while ORR is 35% (95% CI; 21–50) [61]. High PFS and DCR suggest that zanidatamab is highly effective in the stability of breast malignancy while modest ORR suggests it does not always lead to shrinkage of tumors since it significantly shrinks tumors in 37% of patients.

Lumish et al. (NCT04513665) investigated zanidatamab for endometrial malignancies and recorded PFS 6.2 months and a low ORR (6.2%) which may be due to the downregulation of HER2 expression [50]. The reason could be the adaptation of the tumor due to earlier treatment of patients by anti-HER2 receptor drugs making it less responsive to zanidatamab. Therefore, more trials should be conducted evaluating this drug for endometrial cancer since there are no ongoing studies for this purpose.

## Discussion

This narrative review aimed to assess the efficacy of zanidatamab in several types of HER2-positive malignant tumors. Our review of trials reveals remarkable outcomes of ORR, PFS, and DCR. However, zanidatamab is not yet FDA-approved but has been accepted and granted priority review of the BLA for the treatment of previously treated, unresectable, locally advanced, or metastatic HER2-positive BTC. FDA has set a target action date of November 29, 2024 [37]. The FDA has not granted priority review of the BLA for any other malignancy treatment by zanidatamab.

In light of the above-mentioned trials, we can conclude that zanidatamab is highly effective for BTC, breast cancers, GEA, and NSCLC with a significant disease control rate. It produced strong ORRs between 35% and 90.9% for a variety of malignancies, including biliary tract, colorectal, and breast cancers, and DCRs between 69 and 100% in several studies. The PFS ranged from 6.2 to 16.4 months, which is noteworthy. Although zanidatamab showed promise in patients who had received extensive pretreatment, the results for endometrial cancer were less encouraging, suggesting that more research is necessary in that area given the poor ORR and PFS outcomes.

There are ongoing clinical trials for each malignancy mentioned above which could provide beneficial evidence which can lead to its FDA approval. However, there is no trial evaluating the efficacy of zanidatamab for NSCLC.

The results of HERIZON-BTC-01 support that zanidatamab is potent as a treatment choice in HER2-positive BTC. In this trial 18% of patients had grade 3 TRAE; the most common were diarrhea and decreased ejection fraction. There were no grade 4 TRAE and no treatment-related deaths [44]. Compared to the adverse effects reported for Pertuzumab plus trastuzumab (FDA-approved dual anti-HER2 regimen) which were reported in 46% of patients with Grade 3–4 TRAE, the most common is increased alanine aminotransferase and aspartate aminotransferase. Serious TRAE was seen in 26% of patients. There were no treatment-related deaths [62]. Zanidatamab demonstrated meaningful clinical benefit with a manageable safety profile in patients with treatment-refractory, HER2-positive BTC. A study that was done on various HER2-amplified cancers with zanidatamab found that the most frequent TRAE were diarrhea and infusion reactions. There were no treatment-related deaths in any clinical trials [44].

Zanidatamab elicits many actions greater than trastuzumab due to its different mechanisms of action. Zanidatamab has shown promising results in many HER2-amplified cancers in many clinical trials, but its long-term safety and efficacy are yet to be evaluated through ongoing trials mentioned in Tables 5 and 6. As these are randomized controlled trials their results may not translate into real-life settings because these are done in a highly controlled environment, but with the time and pending results from the ongoing trials will help us decide the drug's long-term safety.

While Phase 3 clinical study is still underway to determine broader efficacy and adverse events [51], studies conducted thus far have shown consistent and reproducible results between Phase 1 and Phase 2 clinical trials. Research has proven to be highly effective and has shown a good safety record in patients with cancerous tumors that express HER2 receptors [44, 49, 58, 63]. The most common adverse event reported is diarrhea, followed by allergic reactions to the drug. Few patients developed cardiac issues with symptoms consistent with HER2 receptor inhibition. Almost all symptoms were non-severe and classified as Group 1 and 2 events, requiring only outpatient management [44, 49, 58, 63]. Research has shown a good efficacy of the treatment with studies documenting the first confirmed response at 1.8 months for HER2-positive malignant tumors. It has shown a good disease control rate and reduced measured tumor burden in most cases. The average time of response is seen at 12.9 months. It has also shown positive median PFS and OS during the studies [44, 49, 58]. It has been shown to improve the quality of life in most patients [63]. This drug has gained significant approval from the FDA, having already obtained the special designation for significant advancement in therapy for BTC with HER2 gene amplification that has been previously treated. Moreover, it has received two Fast Track recognitions, one for previously treated or recurrent HER2-positive BTC, and another for the initial therapy of GEA alongside standard chemotherapy. Furthermore, it has been granted Orphan Drug status by the FDA for treating BTC and gastric cancers, as well as for gastric cancer by the EMA [64].

Research on Zanidatamab's effectiveness in other HER2-positive cancer types is ongoing. Endometrial carcinoma, BTC, and HER2-positive breast cancer are the main topics of the current data. Additional research is required to fully comprehend the drug's potential in additional HER2-positive solid tumors, including less prevalent ones. Combinations with docetaxel and other unidentified chemotherapies are being investigated in the present investigations. There is a need to perform more thorough combination studies to determine the best combination treatments that can boost Zanidatamab's effectiveness. In the next five years, more research is likely to unfold Zanidatamab's long-term safety and effectiveness, particularly concerning OS. Response rates and progression-free survival are the main topics of the available data.

Zanidatamb shows better efficacy than trastuzumab due to its alternative mechanisms. In many clinical trials, Zanidatamab has demonstrated potential outcomes in many HER2-amplified cancers. To obtain more information on the long-term results, including OS, for patients treated with zanidatamab, the current trials should extend their follow-up periods [44]. Based on current results, zanidatamab is likely to continue its development and may obtain regulatory approvals for the treatment of HER2-positive cancers, particularly in combination with other therapies. Ongoing and future clinical trials will enhance the understanding of zanidatamab’s efficacy and safety across a broader range of HER2-positive cancer types. If long-term studies confirm sustained responsiveness, zanidatamab could become an important addition to the treatment options for HER2-positive cancers.

There is a need to investigate the resistance mechanisms to zanidatamab, including the amplification or overexpression of HER2, immune evasion mechanisms, and the activation of alternative signaling pathways. Understanding these resistance mechanisms could inform the development of strategies to overcome or prevent resistance in the future, such as determining which combination therapy alongside zanidatamab can effectively address this issue. Currently, there are limited studies available regarding its use in ovarian cancer, NSCLC, and colorectal cancer. Therefore, more clinical trials should be conducted on these types of cancer to evaluate the effectiveness of zanidatamab.

Zanidatamab is primarily assessed in patients with advanced-stage malignancies, and none of the trials have focused on its use in early-stage cancers. More research is needed to explore the efficacy of zanidatamab in these earlier stages.

In HER2-positive cancers, identifying biomarkers can help predict how patients will respond to zanidatamab. Studies have shown that changes in the PI3K/AKT pathway, including PIK3CA mutations and PTEN loss, can influence the effectiveness of HER2-targeted therapies. For instance, a study published in Biomarker Research highlights how mutations that activate the PI3K pathway, such as PIK3CA mutations and PTEN loss, can impact the efficacy of HER2-targeted treatments. [65].

Additionally, treatment outcomes may be influenced by variations in HER2 expression within tumors. The mechanism by which zanidatamab binds to adjacent HER2 molecules and induces specific HER2 rearrangements is discussed in an article published in Nature Communications. [66]. This could have implications for tumors with variable HER2 expression. While there are limited direct studies on this topic, the findings suggest that evaluating HER2 expression levels and changes in the PI3K/AKT pathway may help identify patients who would benefit the most from zanidatamab therapy.

## Strengths, Limitations, and Future Prospects

Zanidatamab has demonstrated promising efficacy against various solid tumors. This represents a departure from conventional treatments that primarily rely on sequential chemotherapies until disease progression. Various clinical trials investigating zanidatamab produced strong ORR, DCR, and PFS for various malignancies, including biliary tract, colorectal, gastroesophageal, breast, ovarian, and non-small cell lung carcinomas. Although zanidatamab showed promise in patients who had received extensive pretreatment, the results for endometrial cancer were less encouraging, suggesting that more research is necessary in that area given the poor ORR and PFS outcomes.

The bispecific design of zanidatamab offers a novel mechanism of action, potentially enhancing treatment outcomes by combining immune-mediated cytotoxicity with dual HER2 inhibition. However, the current lack of reliable long-term safety and efficacy data is a significant limitation, as most studies are still in their early phases. Therefore, there is an urgent need for well-designed prospective clinical trials using standardized assessment methodologies to better understand its long-term effectiveness, safety profile, and optimal application across different HER2-positive malignancies. Neoadjuvant and adjuvant therapies are integral to comprehensive cancer treatment strategies, aimed at reducing cancer recurrence and improving survival rates. Further research and expanded treatment approaches hold substantial promises for advancing care and outcomes in challenging cancers like those involving HER2 positivity. Resistance to HER2-targeted treatments, driven by factors such as HER2 mutations or alternative signaling pathways, remains a critical issue. Investigating zanidatamab's potential to bypass or overcome these resistance mechanisms is crucial for its broader clinical success. Compared to trastuzumab, a monospecific antibody, preclinical studies have shown zanidatamab to be more effective. Early-stage clinical trials combining zanidatamab with chemotherapy have reported strong anticancer activity and a manageable safety profile. Zanidatamab with chemotherapy has shown excellent anticancer activity with a tolerable safety profile in early-stage clinical trials.

## Conclusion

In summary, while zanidatamab shows considerable potential in treating HER2-positive cancers, ongoing research is essential to establish its long-term benefits, address resistance mechanisms, and optimize its role in improving outcomes for patients facing these difficult-to-treat malignancies.

## Supplementary Information

Supplementary Material 1.

## Data Availability

No datasets were generated or analysed during the current study.

## Abbreviations

HER2: Human Epidermal Growth Factor Receptor 2
EGFR: Epidermal Growth Factor Receptor
ERBB2: Erythroblastic oncogene B gene
BTC: Biliary Tract Cancer
IHC: Immunohistochemistry
NGS: Next-Generation Sequencing
FISH: Fluorescent In-Situ Hybridization
CTC: Circulating Tumor Cells
CTD: Circulating Tumor DNA
BLA: Biologics License Application
PDUFA: Prescription Drug User Fee Act
CTL: Cytotoxic T-Lymphocyte
ADCC: Antibody-Dependent Cell-Mediated Cytotoxicity
ADCP: Antibody-Dependent Cellular Phagocytosis
TRAE: Treatment-Related Adverse Events
IHCh: Intra-hepatic Cholangiocarcinoma
ECC: Extra-hepatic Cholangiocarcinoma
GBC: Gall Bladder Cancer
NSCLC: Non-Small Cell Lung Carcinoma
DLTs: Dose Limiting Toxicities
ORR: Objective response rate
CBR: Clinical Benefit Rate
DCR: Disease Control Rate
DoR: Duration of Response
PFS: Progression-Free Survival
OS: Overall Survival
pCR: Pathologic Complete Response
NR: Not Reported
NYR: Not Yet Recorded
