# Supplementary material for: Exploring Zanidatamab’s efficacy across HER2-positive Malignancies: a narrative review
Source: BMC Cancer. 2025 Mar 1;25:382. doi: 10.1186/s12885-025-13749-1 (PMC11871714; doi:10.1186/s12885-025-13749-1)
Supplement: Supplementary file 1 — Supplementary Material 1. [file 12885_2025_13749_MOESM1_ESM.docx]

**Identification of studies via other methods**

**Identification of studies via databases and registers**

Records identified from:

Websites (n = 5 )

Organisations (n = 0)

Citation searching (n = 0)

etc.

Records removed *before screening*:

Duplicate records removed (n =10 )

Records marked as ineligible by automation tools (n =0 )

Records removed for other reasons (n =0 )

Records identified from*:

Databases (n =2 )

Pubmed (n=12)

Cochrane library (n=10 )

Registers (n =1 )

Clinicaltrails.gov (n= 9)

**Identification**

Records screened

(n =21 )

Records excluded**

(n =10)

Reports not retrieved

(n = 0)

Reports sought for retrieval

(n =5 )

Reports sought for retrieval

(n =11 )

Reports not retrieved

(n =0)

**Screening**

Reports assessed for eligibility

(n =5 )

Reports excluded:

(n= 0)

Reports assessed for eligibility

(n =11)

Reports excluded:

(n =0)

Studies included in review

(n =16)

**Included**

*Consider, if feasible to do so, reporting the number of records identified from each database or register searched (rather than the total number across all databases/registers).

**If automation tools were used, indicate how many records were excluded by a human and how many were excluded by automation tools.

Source: Page MJ, et al. BMJ 2021;372:n71. doi: 10.1136/bmj.n71.

This work is licensed under CC BY 4.0. To view a copy of this license, visit <https://creativecommons.org/licenses/by/4.0/>
